# Assessment of Extraction Parameters on Antioxidant Capacity, Polyphenol Content, Epigallocatechin Gallate (EGCG), Epicatechin Gallate (ECG) and Iriflophenone 3-C-*β*-Glucoside of Agarwood (*Aquilaria crassna*) Young Leaves

**DOI:** 10.3390/molecules190812304

**Published:** 2014-08-14

**Authors:** Pei Yin Tay, Chin Ping Tan, Faridah Abas, Hip Seng Yim, Chun Wai Ho

**Affiliations:** 1 Department of Food Technology, Faculty of Food Science and Technology, Universiti Putra Malaysia, 43400 Serdang, Malaysia; E-Mail: peiyin_tay@hotmail.com; 2 Department of Food Science, Faculty of Food Science and Technology, Universiti Putra Malaysia, 43400 Serdang, Malaysia; E-Mail: faridah_abas@upm.edu.my; 3 Department of Food Science and Nutrition, Faculty of Applied Sciences, UCSI University, No. 1, Jalan Menara Gading, UCSI Heights, Cheras, 56000 Kuala Lumpur, Malaysia; E-Mail: hsyim@ucsi.edu.my

**Keywords:** agarwood (*Aquilaria crassna*) leaves, epigallocatechin gallate (EGCG), epicatechin gallate (ECG), iriflophenone 3-C-*β*-glucoside, polyphenol, antioxidant capacity

## Abstract

The effects of ethanol concentration (0%–100%, v/v), solid-to-solvent ratio (1:10–1:60, w/v) and extraction time (30–180 min) on the extraction of polyphenols from agarwood (*Aquilaria crassna*) were examined. Total phenolic content (TPC), total flavonoid content (TFC) and total flavanol (TF) assays and HPLC-DAD were used for the determination and quantification of polyphenols, flavanol gallates (epigallocatechin gallate—EGCG and epicatechin gallate—ECG) and a benzophenone (iriflophenone 3-C-*β*-glucoside) from the crude polyphenol extract (CPE) of *A. crassna*. 2,2'-Diphenyl-1-picrylhydrazyl (DPPH) radical scavenging activity was used to evaluate the antioxidant capacity of the CPE. Experimental results concluded that ethanol concentration and solid-to-solvent ratio had significant effects (*p <* 0.05) on the yields of polyphenol and antioxidant capacity. Extraction time had an insignificant influence on the recovery of EGCG, ECG and iriflophenone 3-C-*β*-glucoside, as well as radical scavenging capacity from the CPE. The extraction parameters that exhibited maximum yields were 40% (v/v) ethanol, 1:60 (w/v) for 30 min where the TPC, TFC, TF, DPPH, EGCG, ECG and iriflophenone 3-C-*β*-glucoside levels achieved were 183.5 mg GAE/g DW, 249.0 mg QE/g DW, 4.9 mg CE/g DW, 93.7%, 29.1 mg EGCG/g DW, 44.3 mg ECG/g DW and 39.9 mg iriflophenone 3-C-*β*-glucoside/g DW respectively. The IC_50_ of the CPE was 24.6 mg/L.

## 1. Introduction

Agriculture by-products are considered as undervalued substrates due to their removal from food production lines and the environmental effects caused by their disposal [1]. Nowadays, with the prospects of feeding a fast growing population in the 21st century, agriculture by-products can be valorised as a source of valuable nutraceuticals and they are identified as the ultimate substrates for the development of new products with market value [2,3]. Oreopoulou and Tzia [4] reported phenols and carotenoids from fruit by-products can be applied as natural food preservatives since they extend the shelf-life of products by delaying the formation of off-flavours and rancidity. Pectin can be utilized as gelling agent in confectionary or as fat replacement in meat products [3].

During the cultivation of agarwood (*Aquilaria crassna*), the young leaves are by-products that are usually discarded or converted into low value fertiliser. Agarwood has been used as a sedative, analgesic and digestive in traditional medicine; high attention is focused on its oil and resin that are widely used in perfumes, incenses and as an ingredient in medical recipes [5,6]. Agarwood has been widely cultivated in south and south-east Asia, including Bangladesh, Bhutan, India, Indonesia, Iran, Myanmar, Philippines, Singapore, Thailand and Malaysia [7]. In Malaysia, some 1000 hectares of agarwood were planted a decade ago [8]. Consequently, the wide availability of agarwood ensures an easily accessible and sustainable source of its by-product, young leaves.

Today, there is abundant evidence suggesting that phytochemicals from natural plant sources possess antioxidant properties and these compounds are associated with a lower risk of mortality from many diseases [9,10,11]. The most important group of bioactive compounds from plant sources are the polyphenols, which are mainly made up of flavonoids [12]. Flavonoids have been reported to possess anti-inflammatory, anti-allergic, anti-viral, and anti-proliferative activities and to protect against many chronic diseases, including cancer and cardiovascular disorders [12,13].

An effective solvent extraction system is a good way to achieve cost efficiency and ensure the economic feasibility in an industrial process. Many parameters have been established to affect the efficiency of the solvent extraction process, including extraction methods, type of solvent, solid-to-solvent ratio, extraction time, extraction temperature and pH [13,14]. Among the various factors, solvent concentration, solid-to-solvent ratio and extraction time receive high attention and are often evaluated. The polarity of the solvent is a crucial parameter in the selective extraction of different flavonoid families [15,16]. The solid-to-solvent ratio affects the yield and recovery of antioxidant compounds [17,18], and the extraction time play an important role in minimising energy usage and the cost associated with any extraction process [19]. As a result, this study was aimed at investigating the effects of ethanol concentration (0%–100%), solid-to-solvent ratio (1:10–1:60, w/v) and extraction time (30–180 min) on the extraction of polyphenols from *A. crassna*.

## 2. Results and Discussion

### 2.1. Ethanol Concentration Evaluation

#### 2.1.1. Polyphenol Content of the CPE of *Aquilaria crassna*

**Figure 1:**
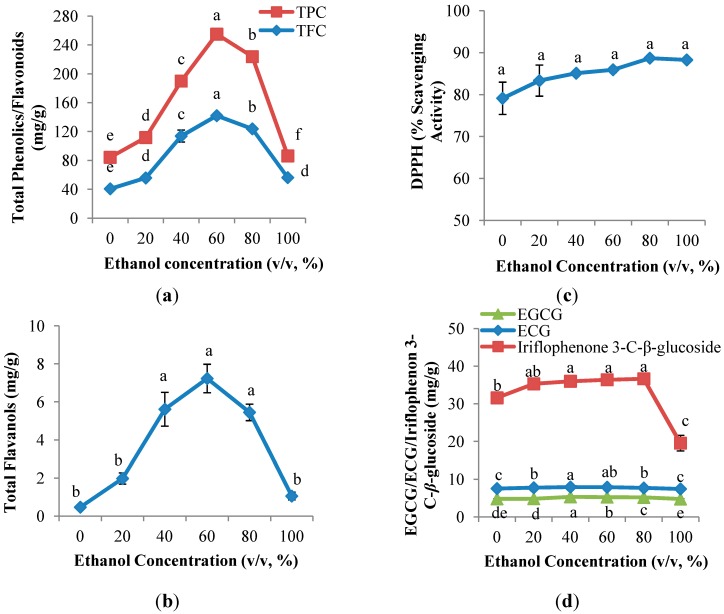
Effect of ethanol concentration on (**a**) TPC and TFC; (**b**) TF; (**c**) DPPH radical scavenging capacity assay; and (**d**) EGCG, ECG and iriflophenone 3-C-*β*-glucoside of CPE from *A. crassna* (*n* = 6). Values are presented as means ± SD. Values marked with the different lower case letters (a–e) are significantly (*p* < 0.05) different.

In present study, the effect of ethanol concentration on polyphenol yields in a CPE of *Aquilaria crassna* was evaluated (Figure 1). The polyphenol content determined by TPC, TFC and TF assays ranged from 30.1 to 112.9 mg GAE/g DW, 40.7 to 142.0 mg QE/g DW and from 0.5 to 7.2 mg CE/g DW, respectively. Figure 1a,b shows that parabola shapes were observed in the TPC, TFC and TF, in which the yields were increased with the increasing ethanol concentration, up to 60% (v/v), and followed by a decrease.

As previously reported in other studies [19], 60% (v/v) ethanol provides the highest yield of both TPC and TFC in the extraction of phenolic compounds from Pegaga (*Centella asiatica*). Remarkably low recoveries of TPC, TFC and TF were observed at 0% and 100% (v/v) ethanol (Figure 1a,b). In general, a single solvent is not good as an extraction solvent as it gives maximum flavonoid recovery as compared to aqueous ethanol. The extraction of bioactive compounds from dried materials involves two steps; the first step involves swelling and rehydration processes and the second step is the mass transfer of soluble constituents from the material to the solvent by diffusion and osmosis [20]. Water plays an important role in the first step swelling and rehydration processes in which a large number of hydroxyl groups are taken up by the cell wall, leading to swelling, whereas ethanol is responsible for mass transfer of the compounds with a similar polarity, based on the “like-dissolve-like” principle [14,21]. As a result, the low extraction yield of polyphenols using 100% (v/v) ethanol is most likely due to the reason that there was no water contributing to the swelling process, thus resulting in a decrease of contact surface area between the plant matrix and the solvent [13]. In contrast, the low recoveries of polyphenols using 100% (v/v) water could be due to the variety of polyphenols in the *A. crassna* CPE. This is supported by the results of Rebey *et al.* [22], who stated that a binary solvent containing hydro-organic solvents improved the flavonoid extraction yield from cumin seeds.

#### 2.1.2. Antioxidant Capacity of the CPE of *Aquilaria crassna*

As indicated in Figure 1c, the % radical scavenging activity increased insignificantly from 0% (v/v) ethanol (79.1%) and achieved the maximum activity at 100% (v/v) ethanol (88.3%). In contrast to the antioxidant compound assays (TPC, TFC and TF) described in Section 2.1.1, 100% (v/v) ethanol surprisingly exhibited the highest DPPH radical scavenging activity, despite its low polyphenol content. It was known that only polyphenols of a certain structure and particularly hydroxyl position in the molecule determine the antioxidant properties; in general, these properties depend on the ability to donate hydrogen or electron to a free radical [23]. Luís *et al.* [24] reported that there was no correlation between the amount of flavonoids and the antioxidant activity of some Portuguese shrub extracts.

#### 2.1.3. Quantitative and Qualitative Investigation of the CPE of *Aquilaria crassna*

Effects of ethanol concentration on the recovery of flavanol gallates (EGCG and ECG) and benzophenone (iriflophenone 3-C-*β*-glucoside) is shown in Figure 1d. Parabola shapes were observed in both EGCG and ECG; the yields of EGCG and ECG increased gradually from 0% (v/v) ethanol and achieved the maximum value (5.3 mg EGCG/g DW; 7.9 mg ECG/g DW) at 40% (v/v) ethanol, followed by a continuous decrease. As expected, this result was comparable with that reported in Section 2.1.1, which confirmed that flavonoids in the CPE of *A. crassna* possess the same polarity as 40% (v/v) ethanol. In contrast, the highest yield of iriflophenone 3-C-*β*-glucoside was observed at 80% (v/v) ethanol (36.65 mg iriflophenone 3-C-*β*-glucoside/g DW), which indicated that iriflophenone 3-C-*β*-glucoside is less polar compare to EGCG and ECG.

From 20% to 80% (v/v) ethanol, the yields of EGCG, ECG and iriflophenone 3-C-*β*-glucoside were significantly (*p <* 0.05) lower than that of either 0% or 100% (v/v) ethanol. The use of water as the only solvent yields a CPE with high content of impurities such as organic acids, sugars, and soluble proteins, which can negatively affect phenolic identification and quantification [21]. Moreover, 100% (v/v) ethanol is able to destroy or denature polyphenols [25]. In short, either 100% (v/v) ethanol or water are not the best extraction solvents for EGCG, ECG and iriflophenone 3-C-*β*-glucoside.

Even though 60% (v/v) ethanol resulted in the highest yields of antioxidant compounds, including TPC, TFC, TF, and flavanol gallates as well as the highest iriflophenone 3-C-*β*-glucoside at 80% (v/v) ethanol, the DPPH radical scavenging activity was observed to have insignificant differences between 40%, 60% and 80% (v/v) ethanol. Therefore, from an economic perspective, a 40% (v/v) ethanol concentration was eventually chosen for the evaluation of solid-to-solvent ratios.

### 2.2. Solid-to-Solvent Ratio Evaluation

#### 2.2.1. Polyphenol Content of the CPE of *Aquilaria crassna*

**Figure 2:**
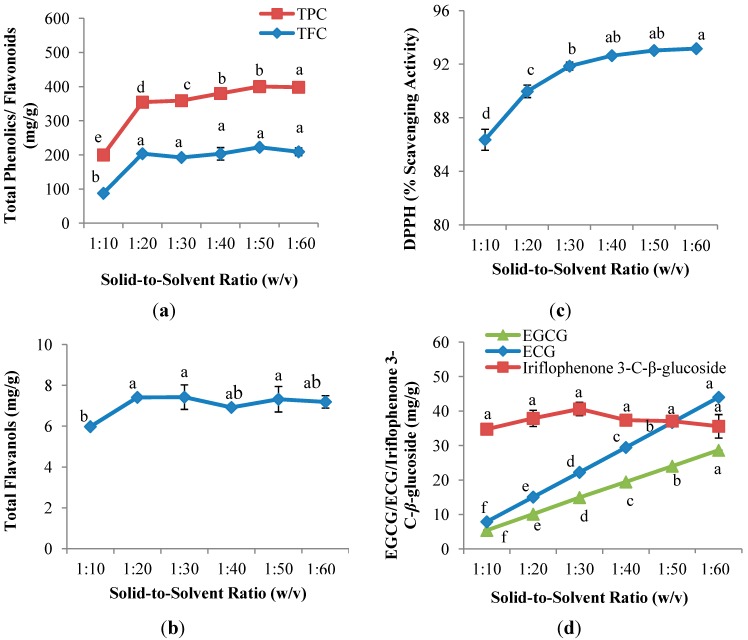
Effect of solid-to-solvent ratio on (**a**) TPC and TFC; (**b**) TF; (**c**) DPPH radical scavenging capacity assay; and (**d**) EGCG, ECG and iriflophenone 3-C-*β*-glucoside of CPE from *A. crassna* (*n* = 6). Values are presented as means ± SD. Values marked with the different lower case letters (a–f) are significantly (*p <* 0.05) different.

Figure 2a,b shows that the solid-to-solvent ratio had significant (*p <* 0.05) influence on the yields of TPC, TFC and TF. The recovery of TPC was observed to increase gradually (*p <* 0.05) from 1:10 (w/v) solid-to-solvent ratio and achieved the highest recovery at 1:60 (w/v). On the other hand, different trend was observed in TFC and TF; the extraction yields of TFC and TF increased significantly (*p <* 0.05) from 1:10 (w/v) to 1:20 (w/v); followed by insignificant increase when the solid-to-solvent ratio increased to 1:60 (w/v). This result suggested that saturation of solvent occurred at 1:20 (w/v) and final equilibrium was achieved [26]. A similar study was reported by Silva *et al.* [27] on the effect of solid-to-solvent ratio of the extraction of phenolics from *I. edulis* leaves; in that study, the yield of phenolics increased from 1:10 (w/v) and reached equilibrium with further increases in the solid-to-solvent ratio (1:20, 1:40 and 1:80; w/v).

#### 2.2.2. Antioxidant Capacity of the CPE of *Aquilaria crassna*

Figure 2c illustrates that the solid-to-solvent ratio had significant (*p <* 0.05) effects on the antioxidant capacity of the CPE of *A. crassna*. DPPH radical scavenging capacity increased gradually from 1:10 (w/v) (86.4%) to 1:60 (w/v) (93.2%). The lowest value of DPPH radical scavenging capacity, 86.4%, occurred at the solid-to-solvent ratio of 1:10 (w/v), followed by an insignificant increase at the ratios of 1:30, 1:40, 1:50 and 1:60 (w/v). Although low flavonoid content was observed at 1:60 (w/v) (Section 2.2.1), it exhibited the highest % DPPH scavenging capacity. This is likely due to the dilution of impurities that may affect negatively on the scavenging activity at high solid-to-solvent ratio [13].

#### 2.2.3. Quantitative and Qualitative Investigation of the CPE of *Aquilaria crassna*

In Figure 2d, it is observed that there was no significant difference on the yield of iriflophenone 3-C-*β*-glucoside with the increase solid-to-solvent ratio. Contradictory results were observed in the recovery of both EGCG and ECG; EGCG and ECG increased significantly (*p <* 0.05) and linearly from solid-to-solvent ratio of 1:10 (w/v) (5.4 mg EGCG/g DW; 7.9 mg ECG/g DW) to 1:60 (w/v) (28.6 mg EGCG/g DW; 44.0 mg ECG/g DW). Mass transfer principles state that the concentration gradient between solid and solvent is the driving force for mass transfer [28]. An increase in solid-to-solvent ratio results in an increase in concentration gradient, which results in an increase in the diffusion rate thus permitting greater extraction of solids by the solvent [17,29]. Zhang *et al.* [30] observed high leaching-out rates of bioactive compounds when the extraction solvent was increased.

Although the values of TFC, TF, DPPH radical scavenging activity and iriflophenone 3-C-*β*-glucoside showed insignificant differences between 1:40 (w/v) and 1:60 (w/v), the yields of TPC at 1:60 (w/v) was significantly (*p* > 0.05) higher as compared to 1:40 (w/v) and the concentration of EGCG and ECG at 1:60 (w/v) was approximately two times higher than 1:40 (w/v). Therefore, 1:60 (w/v) was chosen as the best solid-to-solvent ratio and was used to evaluate the effect of extraction time on all studied parameters.

### 2.3. Extraction Time

#### 2.3.1. Polyphenol Content of the CPE of *Aquilaria crassna*

**Figure 3:**
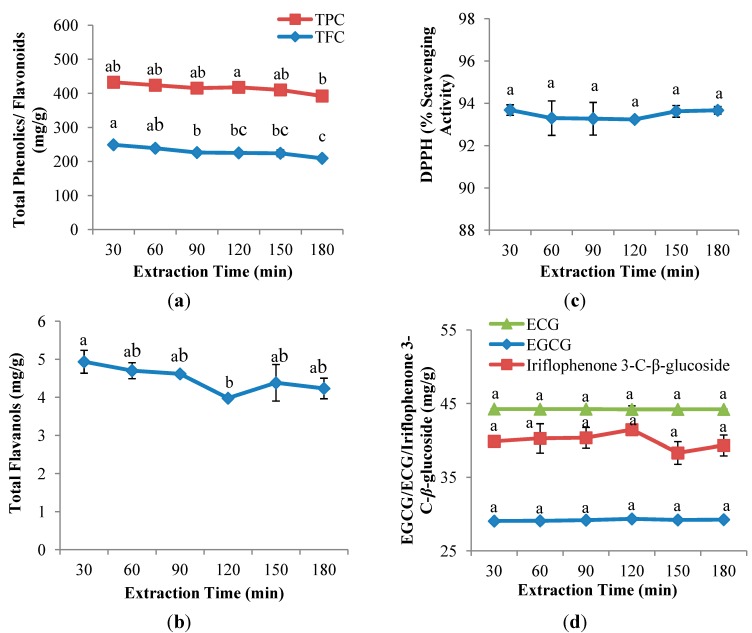
Effect of extraction time on (**a**) TPC and TFC; (**b**) TF; (**c**) DPPH radical scavenging capacity assay; and (**d**) EGCG, ECG and iriflophenone 3-C-*β*-glucoside of CPE from *A. crassna* (*n* = 6). Values are presented as means ± SD. Values marked with the different lower case letters (a–c) are significantly (*p <* 0.05) different.

As Figure 3a,b shows, extraction time (30, 60, 90, 120, 150 and 180 min) had significant (*p <* 0.05) influences in TPC, TFC and TF. The maximum yields of both TFC and TF were achieved at the shortest extraction time, 30 min, with yields of 249.0 mg QE/g DW and 4.9 mg CE/g DW, respectively, whereas, although TPC achieved the highest yield at 120 min (192.6 mg GAE/g DW), no significant different was observed between 120 min and 30 min. As the extraction time increased, both TPC and TFC yields decreased gradually. A prolonged extraction time may cause increased exposure to light and oxygen, which will eventually lead to the oxidation of phenolics unless reducing agents were added to the solvent system [27,31,32]. A similar finding on the effect of extraction time on the extraction of phenolics from *Centella asiatica* was reported by Chew *et al.* [19], who stated that endogenous enzyme in plant tissues have the ability to destroy phenolic compounds when the extraction time increased.

Using the same solvent extraction parameters, TFC and TF showed a different trend with the increased extraction time; TF was observed to decrease and increase insignificantly. According to Herodež *et al.* [33], solvent extraction consists of two stages, including an initial fast step in which the recovery of solutes from the superficial sites of the raw material occurs; followed by a second slower step corresponding to the molecular diffusion of solutes from the internal sites through the porous medium. As a result, the variety of the constituents in natural plants, including different degree of polyphenols polymerisation, solubility of polyphenols and interaction of polyphenols with other food constituents, may lead to the different times being required for the first and second step of solvent extraction [29].

#### 2.3.2. Antioxidant Capacity of the CPE of *Aquilaria crassna*

Extraction time had no significant effect on the antioxidant capacity of CPE from *A. crassna* (Figure 3c). Lapornik *et al.* [25] indicated that the antioxidant activity of water extracts from red and black currant did not change when different extraction times were used. The maximum DPPH radical scavenging capacity of CPE from *A. crassna* was observed at 180 min (93.7%), showing a contrary tendency with TPC, TFC and TF as described in Section 2.3.1. This was also observed in the study by Thoo *et al.* [21], who demonstrated that the measurement of DPPH radical scavenging capacities does not solely depend on a single group of antioxidant compounds, and indeed it is based on the ability of any compound present that can scavenge DPPH radicals.

#### 2.3.3. Quantitative and Qualitative Investigation of the CPE of *Aquilaria crassna*

Figure 3d shows that extraction time had insignificant effects on the recovery of flavanol gallates (EGCG and ECG) and benzophenone (iriflophenone 3-C-*β*-glucoside). The maximum yield of EGCG and ECG was achieved at the shortest extraction time, 30 min (29.1 mg EGCG/g DW; 44.3 mg ECG/g DW) while iriflophenone 3-C-*β*-glucoside was observed at 120 min (41.45 mg iriflophenone 3-C-*β*-glucoside/g DW). As the results suggest, further increases in process duration did not significantly improve the recovery of EGCG and ECG as well as iriflophenone 3-C-*β*-glucoside. A similar study of henna leaves by Uma *et al.* [34], revealed that there was no significant difference in the extraction of phenolic compounds between a longer (450 min) and a shorter extraction time (90 min).

In present study, the highest values in assays (TFC, TF, DPPH radical scavenging activity and flavanol gallates) were observed at 30 min and no significant difference was found between 30 min and 120 min on the recovery of TPC and iriflophenone 3-C-*β*-glucoside. Therefore, 30 min was chosen as an optimum extraction time.

### 2.4. Comparative Study with Reference Antioxidants

The IC_50_ of CPE of *A. crassna* was 24.6 mg/L, significantly (*p* < 0.05) different from both butylated hydroxyanisole (BHA) and (+)-catechin (13.6 mg/L and 11.7 mg/L). BHA and (+)-catechin possessed higher antioxidant capabilities than the CPE of *A. crassna*. Low antioxidant activity of the CPE of *A. crassna* could be due to the loss of polyphenols that are responsible for antioxidant activity during the processes such as drying, extraction and filtration. Kyi *et al.* [35] reported that the drying process could cause enzymatic oxidation of polyphenols and consequently, resulted in a decline of the concentration of total polyphenols. In addition, extraction conditions could favour the formation of phenol polymers that have the ability to inhibit antioxidant activity [18,36]. In conclusion, even though there are many factors that contributed to the low antioxidant activity of the CPE of *A. crassna*, the IC_50_ of the CPE of *A. crassna* was approximately two times greater than BHA and (+)-catechin.

## 3. Experimental Section

### 3.1. Plant Material

Approximately 5 kg of agarwood (*Aquilaria crassna*) young leaves (first five youngest leaves; yellowish green) was freshly plucked from a plantation in Lenggeng, Negeri Sembilan, Malaysia (Latitude: 2.82, Longitude: 101.88). Fresh young leaves of uniform shape and color were selected; blemished and diseased young leaves were eliminated.

### 3.2. Chemical Reagents

The solvents and reagents used in assays, including TFC, TFA and DPPH radical scavenging capacity assay were of analytical reagent (AR) grade. The solvents and reagents used in reversed phase high performance liquid chromatography (with diode array detector, RP HPLC-DAD) were of HPLC grade. DPPH (95% purity), 4-dimethylaminocinnamaldehyde (DMACA), (+)-catechin hydrate (≥98% purity), sodium nitrite, gallic acid (97% purity), EGCG (≥80% purity), ECG (99.9% purity), BHA and quercetin (≥98% purity) were purchased from Sigma-Aldrich (Steinheim, Germany). Folin-Ciocalteu’s reagent, methanol (AR grade) and methanol (HPLC grade) were purchased from Merck (Darmstadt, Germany). Ethanol (95%, v/v) and sodium carbonate anhydrous were purchased from John Kollin Corporation (Midlothian, UK). Sodium nitrite, sodium hydroxide and aluminum chloride hexahydrate were purchased from Friendemann Schmidt (Schmidt, Germany). Deionised water used throughout the experiments was purified with a Milli-Q water purification system (Millipore Corporation, Billerica, MA, USA).

### 3.3. Sample Preparation

Agarwood (*A. crassna*) fresh young leaves were plucked, sorted, and oven-dried evenly at 50 °C for 20 h in a convection oven (Model UNB 400, Memmert, Schwabach, Germany). After that, dried leaves were ground with a microfine grinding mill at 4000 rpm to obtain fine powder (0.5 mm) (MF 10 B, IKA Werke, Stafen im Breisgau, Germany). Six 15 g batches of dried-ground samples were then vacuum packaged into nylon-linear low density polyethylene (nylon-LDPE) pouches (Flexoprint, Puchong, Malaysia) using a vacuum packager (DZQ400/500, Clarity, Beijing, China). The well-packaged samples were eventually stored in a sealed container at ambient temperature (25 °C) before solvent extraction.

### 3.4. Solvent Extraction

Two grams of agarwood (*A. crassna*) dry powder was extracted with 20 mL of extracting solvents in a conical flask. The conical flask was wrapped with parafilm (Pechiney Plastic Packaging, Chicago, IL, USA) and aluminum foil (Diamond, New York, NY, USA) in order to prevent spilling of the mixture and light exposure, respectively. The mixture was subsequently shaken with a shaker (SK-300, JEIO TECH, Seoul, Korea) at a constant shaking speed of 150 rpm. After extraction, the *A. crassna* extract was subjected to centrifugation at 1957× *g* for 15 min at 20 °C in a centrifuge (Allegra X-22R Centrifuge, Beckman Coulter, Krefeld, Germany) and then filtered through Whatman No. 1 filter paper (Whatman International, Maidstone, England) to obtain a clear filtrate or crude polyphenol extract (CPE). The CPE of *A. crassna* was eventually collected in a light-protected amber bottle for analysis without storage. All extractions were performed as replicates.

### 3.5. Experimental Design

Experiments were performed by a one-factor-at-a-time approach to determine the best conditions (solvent concentration, solid-to-solvent ratio and extraction time) for polyphenols extraction from *A. crassna*. After solvent extraction, the CPE was then subjected to antioxidant assays, namely TPC assay, TFC assay, TF and an antioxidant capacity assay, a DPPH radical scavenging capacity assay. A RP HPLC-DAD was performed to determine two flavanol gallates, EGCG and ECG, as well as one benzophenone, iriflophenone 3-C-*β*-glucoside in the CPE of *A. crassna*.

#### 3.5.1. Ethanol Concentration

Samples were extracted with six different concentration of ethanol ranging from 0% to 100% (v/v) at a solid-to-solvent ratio of 1:10 (w/v) for 180 min. The extraction procedures were described in Section 3.4. The best ethanol concentration was selected in relation to the values of TPC (mg gallic acid equivalent, GAE/g dry weight, DW); TFC (mg quercetin equivalent, QE/g DW); TF (mg catechin equivalent, CE/g DW); DPPH radical scavenging capacity assay (% scavenging activity) and RP HPLC-DAD (mg of individual catechin and benzophenone/g DW).

#### 3.5.2. Solid-to-Solvent Ratio

Using the best ethanol concentration selected from Section 3.5.1 and by fixing an extraction time of 180 min, samples were extracted with six different solid-to-solvent ratios (1:10, 1:20, 1:30, 1:40, 1:50 and 1:60) (w/v). The optimum solid-to-solvent ratio was selected according to the values of TPC (mg GAE/g DW); TFC (mg QE/g DW); TF (mg CE/g DW); DPPH radical scavenging capacity assay (% scavenging activity) and RP HPLC-DAD (mg of individual catechin and benzophenone/g DW).

#### 3.5.3. Extraction Time

Samples were extracted using the best ethanol concentration and the best solid-to-solvent ratio selected from Section 3.5.1 and Section 3.5.2, respectively. The extraction procedures were repeated as described in Section 3.4 by varying the extraction time from 30 to 180 min. The best extraction time was selected based on the value of TPC (mg GAE/g DW); TFC (mg QE/g DW); TF (mg CE/g DW); DPPH radical scavenging capacity assay (% scavenging activity) and RP HPLC-DAD (mg of individual catechin and benzophenone/g DW).

### 3.6. Comparative Study with Reference Antioxidants

#### 3.6.1. Preparation of the IC_50_ (50% Inhibitory Concentration) Curve of the CPE from *A. crassna*

*A. crassna* dried powder was extracted by using the extraction conditions (ethanol concentration, solid-to-solvent ratio and extraction time) that achieved maximum yields of polyphenols. The CPE obtained was subsequently subjected to rotary evaporation (Rotavapor R-200; Heating Bath B-490, Büchi, Hessigkofen, Switzerland) at level 5 and 50 °C for 15 min. The CPE slurry was then dried for 15 h with a freeze-dryer (Alpha 1-4 lo plus, Christ, Grünwald, Germany).

In order to prepare the IC_50_ curve for CPE of *A. crassna*, a 10,000 mg/L stock solution was prepared by first dissolving 0.1 g of dried CPE in 10 mL of the best ethanol concentration selected in the single factor experiments. The stock solution was then diluted to five different concentrations (100, 200, 400, 800 and 1600 mg/L), and each solution was subjected to the DPPH radical scavenging capacity assay. The obtained % scavenging activity of CPE was plotted against the concentration of CPE, and the IC_50_ was obtained by substituting y = 50 into the equation y = 0.0439x + 3.8119 (*R^2^* = 0.996).

#### 3.6.2. Preparation of IC_50_ Curves for Reference Antioxidants

The IC_50_ curves for BHA and (+)-catechin were obtained by firstly preparing a 10,000 mg/L stock solutions for BHA and (+)-catechin. Both stock solutions were diluted to five different concentrations (100, 200, 400, 800 and 1,600 mg/L) and each concentration was measured for its DPPH radical scavenging activity. The IC_50_ calculations were based on the equation obtained from the IC_50_ curves of BHA and (+)-catechin, which were y = 0.0801x + 7.1275 (*R^2^* = 0.99) and y = 0.0965x + 5.9742 (*R^2^* = 0.99), respectively.

### 3.7. Evaluation of Antioxidant Compounds

#### 3.7.1. Determination of Total Phenolic Content (TPC)

Total phenolic content (TPC) was determined using the Folin-Ciocalteu (F-C) assay according to the method described by Zhang *et al.* [37] with slight modifications. Crude extracts obtained from extraction were diluted 40 times before use. Approximately 0.32 mL of diluted samples were placed in aluminum foil-wrapped test tubes (15 mL) followed by 1.6 mL of Folin-Ciocalteu’s reagent (10-fold dilution). After 5 min, 7.5% (w/v) sodium carbonate (1.28 mL) was added. The blank was prepared by replacing 0.32 mL of sample with 0.32 mL of deionized water. The test tubes were mixed with a vortex mixer (VTX-3000L, LMS, Tokyo, Japan) for 5 s before allowed to stand in dark at room temperature for 30 min. Absorbance was measured against the blank at 765 nm using UV-Vis spectrophotometer (UviLine 9400, Secomam, Alès, France). TPC was calculated based on gallic acid calibration curve described by the equation y = 0.0039x + 0.0638 (*R^2^* = 0.99) and expressed as gallic acid equivalents (GAEs) in milligram per g dry weight (mg GAE/g DW).

#### 3.7.2. Determination of Total Flavonoid Content (TFC)

The total flavonoid content (TFC) of the 40-fold diluted CPE of *A. crassna* was investigated according to the procedures described by Thoo *et al.* [21]. A 0.25 mL aliquot of diluted CPE was mixed with deionized water (1.25 mL) and 5% (w/v) sodium nitrite (NaNO_2_, 75 µL). After 6 min, 10% (w/v) aluminum chloride (AlCl_3_, 150 µL) was added, followed by the addition of 1 M sodium hydroxide (NaOH, 0.5 mL). Shortly after the addition of NaOH, the absorbance of the mixture was measured at 415 nm by using a UV-Vis spectrophotometer (UviLine 9400, Secomam). A blank was prepared by replacing 0.25 mL of the CPE with an equal amount of extraction solvent. TFC was expressed as quercetin equivalents (QEs) in milligram QE per g of dry weight (mg QE/g DW), and measurements were performed in triplicate.

#### 3.7.3. Total Flavanol (TF) Assay

Total flavanol content was determined by the method described by Wallace and Giusti [38], with slight modifications. The CPE obtained from the extraction was diluted 10-fold before use. A 160 µL aliquot of the diluted crude extract was mixed with 4-dimethylaminocinnnamaldehyde (DMACA, 2.4 mL) solution. The mixture was then mixed thoroughly by vortexing (VTX-3000L, LMS) and allowed incubate at room temperature for 12 min. Subsequently, absorbance of the mixture was measured with a UV-Vis spectrophotometry (XTD 5, Secomam) at 640 nm. The blank was composed of DMACA solution, and calculations of TFA were based on a (+)-catechin calibration curve described by the equation y = 0.0022x + 0.039 (*R^2^* = 0.99). TFA was expressed as catechin equivalents (CEs) in milligram CE per g of dry weight (mg CE/g DW), and the measurements were performed in triplicate.

### 3.8. Evaluation of Antioxidant Capacity

#### DPPH Radical Scavenging Capacity

Antioxidant capacity was determined by measuring the scavenging capacity of the 2, 2'-diphenyl-1-picrylhydrazyl radical (DPPH•) based on the method used by Thoo *et al.* [21], with slight modifications. CPE (100 µL) was added with prepared DPPH solution (3.9 mL) in an aluminium-wrapped test tube. The test tube was immediately covered with Parafilm (Pechiney Plastic Packaging) and mixed with a vortex mixer (VTX-3000L, LMS). After incubating in the dark at room temperature (25 °C) for 30 min, the absorbance of the DPPH solution was measured against a blank of deionised water at 517 nm in a UV-Vis spectrophotometer (UviLine 9400, Secomam). Negative control was prepared by replacing 100 µL of the sample extract with extraction solvent. Absorbance measurements of the crude extracts and negative controls were performed in triplicate. The final results were expressed as a percentage of DPPH radical scavenging activity, which was calculated based on the expression [1 − (A_s_/A_c_) × 100%], where A_s_ and A_c_ represent the absorbances of the crude extract and control, respectively, at 517 nm.

### 3.9. HPLC-DAD Analysis

The crude extract of *A. crassna* was analyzed by HPLC (1200 Series LC Unit, Agilent, Santa Clara, CA, USA) on a 3.5 µm × 150 mm × 2.1 mm reversed phase column (Eclipse Plus C18, Agilent). Spectra were recorded from 190 to 400 nm, and the chromatogram was monitored at 280 nm. Column oven was performed at the temperature of 30 °C. 10 µL of sample was injected during each injection. A combination of isocratic and gradient elution was carried out by varying the proportion of solvent A (water with 0.1% orthophosphoric acid) to solvent B (methanol with 0.1% orthophosphoric acid) at a flow rate of 0.2 mL/min. The mobile phase composition began at 98% solvent A for 5 min, followed by a linear increase in solvent B to 40% at 60 min. Lastly, solvent B decreased back to 2% at 65 min. 

Peak identification was based on the comparison of retention times and UV spectra of the standards and compounds. The quantitative analysis was based on the calibration curves of EGCG, ECG and iriflophenone 3-C-*β*-glucoside, which were described by the equations y = 42.604x − 1985 (*R^2^* = 0.99), y = 28.857x − 2103.4 (*R^2^* = 0.98) and y = 67.016x + 176.5 (*R^2^* = 0.99), respectively. EGCG, ECG and iriflophenone 3-C-*β*-glucoside were expressed in milligram EGCG per g of dry weight (mg EGCG/g DW) and milligram ECG per g of dry weight (mg ECG/g DW), milligram iriflophenone 3-C-*β*-glucoside per g of dry weight (mg iriflophenone 3-C-*β*-glucoside/g DW), respectively.

### 3.10. Statistical Analysis

The experimental results in this study were analyzed with Minitab software (Minitab Version 15.1.10.). All values were expressed as the mean ± standard deviation (SD) of triplicate measurements of replicate extractions. One-way analysis of variance (ANOVA) with Tukey’s test was used to determine significant differences (*p* < 0.05) between means. 

## 4. Conclusions

The best solvent extraction system that exhibited maximum recovery of polyphenols from *Aquilaria crassna* was determined in this study. The experimental results revealed that ethanol concentration had significant effects (*p <* 0.05) on the yields of TPC, TFC, TF, DPPH radical scavenging capacity, flavanol gallates (EGCG and ECG) and iriflophenone 3-C-*β*-glucoside of *A. crassna*. Nonetheless, no significant effects was observed in TFC and iriflophenone 3-C-*β*-glucoside when different solid-to-solvent ratios were used. Extraction time did not affect DPPH radical scavenging capacity, or the yields of flavanol gallate and iriflophenone 3-C-*β*-glucoside. The extraction parameters with maximum recovery were 40% (v/v) ethanol, 1:60 (w/v) solid-to-solvent ratio and 30 min extraction time; these conditions represented a compromise of the yield of polyphenols (TPC, 183.5 mg GAE/g DW; TFC, 249.0 mg QE/g DW and TF, 4.9 mg CE/g DW), antioxidant capacities (DPPH radical scavenging capacity assay, 93.7%), flavanol gallates (EGCG, 29.1 mg EGCG/g DW and ECG, 44.3 mg ECG/g DW) and iriflophenone 3-C-*β*-glucoside (39.9 mg iriflophenone 3-C-*β*-glucoside/g DW). The antioxidant activity of CPE from *A. crassna* was significantly (*p <* 0.05) different from either BHA or (+)-catechin; the IC_50_ of the CPE of *A. crassna* (24.6 mg/L) was approximately two times greater than BHA (13.6 mg/L) and (+)-catechin (11.7 mg/L).
